# Editorial: Online Psychology Beyond Addiction and Gaming: A Global Look at Mental Health and Internet-Related Technologies

**DOI:** 10.3389/fpsyg.2021.815013

**Published:** 2021-12-16

**Authors:** Elias Aboujaoude, Daria Joanna Kuss, Mike Z. Yao, Louis W. Leung

**Affiliations:** ^1^Department of Psychiatry and Behavioral Sciences, School of Medicine, Stanford University, Stanford, CA, United States; ^2^Psychology Department, Nottingham Trent University, Nottingham, United Kingdom; ^3^College of Media, University of Illinois at Urbana-Champaign, Champaign, IL, United States; ^4^Department of Journalism and Communications, Hong Kong Shue Yan University, Hong Kong, Hong Kong SAR, China

**Keywords:** internet addiction, FOMO (fear of missing out), phubbing, virtual reality, online privacy, chemsex, ecological momentary assessment, internet gaming

The inclusion in 2018 of “Gaming Disorder” into the 11th revision of the International Classification of Diseases (World Health Organization, 2019) represented a milestone in the decades-long research investigation of the topic. It was also a stark reminder of how little attention has been paid to other highly relevant areas within online psychology. From internet-mediated impulsivity and aggression to the effects of living in a post-privacy age, important dimensions of online psychology have been relatively ignored as the field narrowly focused on gaming (Aboujaoude, 2011; Aboujaoude and Starcevic, 2016). The risk of this narrow focus is 3-fold: (i) users who may not be described as gaming “addicts” under any nosology can feel deceptively immune to online psychological harm; (ii) users can seem deceptively healthy to the mental health professionals trying to diagnose and treat them; and (iii) the limited research scope can complicate meaningful regulation of “Big Tech” by underestimating the negative impact of some of these technologies (Aboujaoude and Gega, 2021; Kuss, 2021). In this special issue, we attempt to widen the aperture beyond “traditional” gaming and the closely linked addiction framework to address crucial themes in online psychology that have received comparatively less attention.

FoMO and “phubbing” are two new additions to the popular lexicon and are the subject of studies in this special issue. FoMO refers to the social-media-fed “fear of missing out,” and has been defined as the anxious feeling “that your peers are doing, in the know about, or in possession of more or something better than you” (Barker, 2016). Phubbing represents the increasingly common practice of snubbing someone in a social setting to concentrate on one's phone instead (Chotpitayasunondh and Douglas, 2016). The study by Li et al. explores how FoMO and smartphone addiction may mediate the impact of affect on sleep quality, showing that negative affect was associated with FoMO and smartphone addiction. Although still relatively new, phubbing has quickly spread, with seemingly universal consequences in terms of psychological distress, as suggested by the study by Blachnio et al. of users in 20 countries.

While many effects of internet-related technologies are cross-cultural, it is important to confirm “locally” and incorporate sociocultural specificities. This is particularly true when it comes to screening and diagnostic tools. Chen et al. do so in a population of Chinese fourth to sixth graders, validating the psychometric properties of three scales that target problematic gaming but also problematic social media and smartphone “app” use. Similarly, Burkauskas et al. confirm in their study the psychometric properties of the Lithuanian version of the nine-item Problematic Internet Use Questionnaire (PIUQ-9) in a sample of Lithuania-based students.

While much research into online psychology has involved young students, “digital natives” and “Generation Z,” older adults have often been ignored. This seriously complicates any claims of “universality” when it comes to online harms and opportunities. Older adults are the target of the study by Liu et al., swhich compared them to college students in terms of “telepresence” and emotional responsiveness vis a vis virtual reality (VR) content. More positive attitudes toward the material were reported by older adults. This adds to the recent literature on the potential benefit from VR among older adults. Among other applications, for example, VR has been used to target mild cognitive impairment (Liao et al., 2020).

Increasingly, technology is seen as both the problem and the solution. Beyond VR being the vehicle for addictive gaming and therapeutic interventions, this is reflected in the interest in the moment-by-moment, *in situ* observation of an individual's phenotypic details via smartphone sensing tools, which promises to improve diagnostics and tailor interventions (Huckvale et al., 2019). This idea is developed in the commentary by Lewczuk et al., which focuses on ecological momentary assessment (EMA) and ecological momentary interventions (EMI). The ideal-world-outcome could be to decrease recall bias, increase validity and deliver between-session interventions in subjects' natural environments.

Other aspects of online life can also be seen as healthy or problematic, depending on the degree of engagement and control over the behavior. The internet has transformed age-old dating and sexual practices, for example, often in enriching ways. It has, however, also facilitated compulsive sexual behavior and risky “chemsex” [using drugs to enhance sex] (Giorgetti et al., 2017), in part *via* geolocalizing tools. This, according to the study by Obarska et al., has contributed to vulnerability to depression, substance use and sleep disorders among excessive users of dating apps in the group of men who have sex with men (MSM) that they studied. The results augment the literature on chemsex (Maxwell et al., 2019) by adding an important mental health dimension.

As with the healthy vs. health-compromising use of dating apps, it can be a “fine line” between the all-consuming nature of gaming in *Gaming Disorder* and among career gamers. The motivations driving professional e-sports players were examined in a study of Hungarian gamers by Banyai et al. Competition, skill development and social motivators predicted career planning for professional players.

Creative approaches are called for to help mitigate some of the negative effects of social media and other apps, including compulsive sex and disordered gaming. The current social media model rests on exploiting users' freely supplied personal data in exchange for keeping platforms free. The acceptability of a model that would protect personal information but charge for social media use was explored in the study by Sindermann et al. Only 21.43% of study participants supported such a model, however. This would support recent survey data showing that the majority of social media users do not understand the privacy risks involved (Hitlin and Rainie, 2019).

One reason for discounting the negative impact of social media may be that we don't know the full extent of the problem. In their paper, Marengo et al. contend that most social media research has focused on only one platform, which can underestimate the deleterious effects of social media overall. Their investigation of the usage of Facebook-owned platforms (Facebook, WhatsApp and Instagram) showed that WhatsApp had the widest reach and that personality traits differed by platform use. The study helps answer the call for the scientific exploration of this understudied but highly popular platform (Jailobaev et al., 2021).

Huge socio-politico-cultural transformations have been attributed to psychological processes unfolding online and on social media. The diverse set of articles in this special issue reflects the richness of the field. Collectively, they shed an important light on some understudied facets of online psychology, although much work remains to be done to fully capture the vastness of the topic and propel it beyond gaming and addiction.

## Author Contributions

EA summarized the main findings from the various submissions and wrote the first draft of the editorial. DK, MY, and LL reviewed, expanded, and edited the draft. All authors contributed to the article and approved the submitted version.

## Conflict of Interest

The authors declare that the research was conducted in the absence of any commercial or financial relationships that could be construed as a potential conflict of interest.

## Publisher's Note

All claims expressed in this article are solely those of the authors and do not necessarily represent those of their affiliated organizations, or those of the publisher, the editors and the reviewers. Any product that may be evaluated in this article, or claim that may be made by its manufacturer, is not guaranteed or endorsed by the publisher.
